# Genetic Composition of Laboratory Stocks of the Self-Fertilizing Fish *Kryptolebias marmoratus*: A Valuable Resource for Experimental Research

**DOI:** 10.1371/journal.pone.0012863

**Published:** 2010-09-22

**Authors:** Andrey Tatarenkov, Brian C. Ring, John F. Elder, David L. Bechler, John C. Avise

**Affiliations:** 1 Department of Ecology and Evolutionary Biology, University of California Irvine, Irvine, California, United States of America; 2 Department of Biology, Valdosta State University, Valdosta, Georgia, United States of America; Louisiana State University, United States of America

## Abstract

The hermaphroditic Mangrove Killifish, *Kryptolebias marmoratus*, is the world's only vertebrate that routinely self-fertilizes. As such, highly inbred and presumably isogenic “clonal” lineages of this androdioecious species have long been maintained in several laboratories and used in a wide variety of experiments that require genetically uniform vertebrate specimens. Here we conduct a genetic inventory of essentially all laboratory stocks of the Mangrove Killifish held worldwide. At 32 microsatellite loci, these stocks proved to show extensive interline differentiation as well as some intraline variation, much of which can be attributed to post-origin *de novo* mutations and/or to the segregation of polymorphisms from wild progenitors. Our genetic findings also document that many of the surveyed laboratory strains are not what they have been labeled, apparently due to the rather frequent mishandling or unintended mixing of various laboratory stocks over the years. Our genetic inventory should help to clarify much of this confusion about the clonal identities and genetic relationships of laboratory lines, and thereby help to rejuvenate interest in *K. marmoratus* as a reliable vertebrate model for experimental research that requires or can capitalize upon “clonal” replicate specimens.

## Introduction

When conducting research on laboratory animals, experimentalists often strive for the following [1]: replicability (low variation between measurements in a given test), repeatability (low variation among replicate tests within a laboratory), and reproducibility (similar outcomes in comparable experiments from different laboratories). In many arenas of biological research, all of these desired properties can be improved by using animals with well-characterized or uniform genetic backgrounds. Such is one rationale for the standard use of highly inbred strains of the mouse (*Mus musculus*) as mammalian models in medical and other research [2]–[4]. More generally, genetically identical individuals (clonemates) can be an especially good source of standardized samples for any experiment in which the research protocol demands that genetic variation among specimens be absent or minimized. Vertebrate animals, including fish, are common research subjects in neurobiology, endocrinology, immunology, developmental biology, aquatic toxicology, and cancer biology [5]. However, natural clonal reproduction in vertebrates is relatively rare [6], and this fact has led researchers to develop artificial techniques and breeding schemes that allow the clonal production of genetically uniform animals in several vertebrate taxa [6].

In nature, clonal or quasi-clonal reproduction occurs in various vertebrate species by any of several mechanisms [6]: constitutive or sporadic parthenogenesis, gynogenesis, hybridogenesis, polyembryony (“twinning”), or multi-generation inbreeding (e.g., via consistent self-fertilization within a hermaphroditic lineage [7]). Although each such process can yield multiple individuals that are genetically identical or nearly so, the mechanisms show key differences. For example, reproduction by self-fertilization, which is known to take place routinely in only one vertebrate species [6], leads to high genetic uniformity only if selfing proceeds for many generations, because outcrossing quickly undoes the intense inbreeding that selfing promotes. Another difference relates to levels of intra-individual heterozygosity (*H*). Individuals in parthenogenetic and gynogenetic taxa have high *H* (because such unisexual vertebrate species invariably had hybrid evolutionary origins), but polyembryonic individuals exhibit *H* values that are typical for sexual reproducers, and individuals that emerge from constitutive selfing are highly homozygous. Another major difference concerns the intergenerational transmission of clonality: parents and progeny are essentially genetically identical under parthenogenesis, gynogenesis, and constitutive selfing, but not so under polyembryony.

Different ways of achieving organismal clonality dictate limitations on how clonemate animals are used in experimental research, and they also necessitate precautions on how “clonal” lineages in the lab are produced, maintained, and named. For example, gynogenetic lineages might incorporate sperm-derived DNA occasionally, and selfing lineages might outcross occasionally and thereby initiate new arrays of distinct genotypic lines. Furthermore, the starting point at which a selfing lineage might be used to produce offspring that are effectively identical genetically should be established and controlled (as has been done for the standard inbred strains of house mice [8], [9]). Moreover, the possibility of *de novo* mutations must always be taken into account during any experiment that employs animals from clonal lines, especially when laboratory stocks have been maintained for many generations.

Following Harrington's [10] discovery of hermaphroditic self-fertilization in the Mangrove Killifish *Kryptolebias marmoratus* (formerly *Rivulus marmoratus* Poey, 1880), this small cyprinodontid fish has attracted the attention of many researchers. Indeed, the unique reproductive biology of *K. marmoratus* and the ease by which its genetic system might be manipulated, coupled with the relative ease of maintaining stock lines in culture, have made this species a model system for a variety of studies: population genetics and the evolution of mating systems [11]–[22], developmental biology [23]–[25], behavior [26]–[32], ecology [33]–[38], ecotoxicology [39]–[44], oncology [45]–[61], physiology [62]–[67], and unisexual biology [11], [68]–[72].

Kallman and Harrington [73] were the first to appreciate that self-fertilizing laboratory strains of the Mangrove Killifish can be highly homozygous and isogenic, and therefore effectively “clonal”. Following the pioneering efforts of Kallman and Harrington [73], several later researchers likewise established or perpetuated laboratory lines that originated from wild-caught specimens of *K. marmoratus*. Many of these researchers rely upon the assumption that each such strain can be clonally propagated in the laboratory [74], [75] simply by isolating a hermaphrodite and its offspring, which supposedly reproduce only by selfing. Many publications refer explicitly to the presumed “clonality” of laboratory lines and conclusions often have been based on the assumption that genetic variation was eliminated by the use of such “clones”, or that the comparisons were being drawn among distinct clonal lineages. However, several factors could compromise the presumed homozygosities and isogenicities of long-maintained laboratory lines, or otherwise cloud these stocks' true genetic identities. These factors include residual heterozygosity (genetic variation retained and/or segregated from variation in the ancestral wild progenitor), post-formational mutations (*de novo* variants that postdate a strain's laboratory origin), intermittent outcrossing within or between strains in the laboratory, and any inadvertent mislabeling or misidentification of the genetic stocks.

Furthermore, the fact that outcrossing and the ensuing segregation of recombinant haplotypes are known to occur occasionally in the laboratory [74], [16], plus the documentation of high outcrossing rates in some natural populations of *K. marmoratus* [12], [17], have raised the distinct possibility that unrecognized genetic variation might have been introduced inadvertently into some laboratory stocks by occasional outcross events (either between pairs of hermaphrodites or between hermaphrodites and males) in this androdioecious species [17]. Another potential complication is that most laboratory lines of Mangrove Killifish were derived from field-caught specimens of unknown genotype and heterozygosity. Thus, many “clonal” stocks in laboratories around the world might actually contain genetically distinct sub-lines for any of the several reasons listed above. Here we present a comparative empirical survey of essentially all *K. marmoratus* laboratory stocks from around the world using a large battery of microsatellite loci. Our goals consist of the following: (1) genetically identify established laboratory stocks, (2) evaluate heterozygosities within these lines, (3) address the origins and genetic relationships among these lines, and (4) provide an accessible database of microsatellite genotypes to standardize all stocks and thereby facilitate future research involving *K. marmoratus*.

## Materials and Methods

### Ethics Statement

The Animal Use Protocol (AUP) for handling fish material described here, AUP-00023-2009, was approved by the Valdosta State University Institutional Animal Care and Use Committee under Animal Welfare Assurance Number A4578-01.

### Fish Samples

Eleven laboratories utilizing *K. marmoratus* were initially contacted in the fall of 2008. Eight of these laboratories provided background information regarding their presumably highly inbred stocks (i.e., “clonal” lines). To obtain biological samples from these stocks, we mailed individually labeled microcentrifuge tubes (containing 400 µl of RNAlater; Ambion, Inc.) to each laboratory. Each collection tube was labeled with a unique laboratory number, stock name, and four replica letters *A*, *B*, *C*, *D* each indicating a different individual fish from a specified stock (e.g. 01-*Hon2-A* refers to laboratory 1, stock *Hon2*, fish *A*). Initially, the total number of requested fish samples was 51 stocks X 4 replicas per stock  = 204 requests, of which 199 samples were successfully received. Sample contents were embryos (26%), whole small fish (32%), or fin clippings (42%).

For the laboratory stocks analyzed in this study, the original locations and years of collection from a wild population were determined from prior publications or by personal communication with the researchers who submitted the samples (Table 1). The original dates of collection ranged from 1991 (for *Cchb* and *50.91*) to 2006 (*Dan06*), implying that the minimum duration of each clone in a laboratory had ranged from 4–19 (mean  = 13) years. The various laboratory strains were descended from wild fish that had been collected in Honduras, Belize, Panama, Cuba, Bahama Islands, various counties in southern Florida, and an unknown locality (for the *Hy* strain only).

**Table 1:** Summary of *Kryptolebias marmoratus* laboratory samples analyzed.

No.	Line	Local (County/Park)	City/State	Year	Lab(s)^a^	Samples
1	*Rhl*	Reckley Hill Pond	San Salvador, Bahamas	2001	1,2,3,4,8	24
2	*SsLL*	Little Lake	San Salvador, Bahamas	2001	1,3	8
3	*Bh6*	Norman's Pond Cay	Exumas, Bahamas	1997	2	4
4	*Slc8E*	Nuclear Power Plant (St. Lucie County)	Florida, USA	1995	1,2,4,8	15
5	*Cchb*	Melbourne Beach (Brevard County)	Florida, USA	1991	2,4	7
6	*Ssh*	Melbourne Beach (Brevard County)	Florida, USA	1995	2	4
7	*Enp02*	Homestead Canal, Flamingo (Everglades National Park)	Florida, USA	2002	2,4	7
8	*Vol*	Mosquito Lagoon, Potato Island (Volusia County)	Florida, USA	1995	1,2,4,6,8	17
9	*Vol02*	Mosquito Lagoon, Potato Island (Volusia County)	Florida, USA	2002	2	4
10	*50.91*	Twin Cayes	Papa Gabriel, Belize	1991	1,5	12
11	*Dan92*	South Pelican Beach	Dangriga, Belize	1992	7	4
12	*Dan2K*	4–5 km South of Pelican Beach	Dangriga, Belize	2000	2,3,4	8
13	*Dan06*	4–5 km South of Pelican Beach	Dangriga, Belize	2006	5	4
14	*Hon2*	Bay Islands	Utila, Honduras	1996	1	4
15	*Hon7*	Bay Islands	Utila, Honduras	1996	1	4
16	*Hon9*	Bay Islands	Utila, Honduras	1996	1,2,4	10
17	*Hon11*	Bay Islands	Utila, Honduras	1996	1	4
18	*R2*	Bay Islands	Roatan, Honduras	2000	2,4	8
19	*PanRS*	Bocas del Toro	Panama	1994	7	4
20	*Gitmo*	Guantanamo Bay	Cuba	2004	2	4
21	*Hy*	ND	ND	2003	3,6	8
	Total					164

a Laboratories of origin:

1, Bechler, Elder, Ring (Valdosta State University, U.S.A.).

2, Taylor (Brevard County Environmentally Endangered Lands Program, Florida, U.S.A.).

3, Orlando (University of Maryland, U.S.A.).

4, Earley (University of Alabama, U.S.A.).

5, Wright (University of Guelph, Canada).

6, Kanamori (Nagoya University, Japan).

7, Sakakura (Nagasaki University, Japan).

8, Hsu (National Taiwan Normal University, Taiwan).

### DNA Isolation

Depending on the contents of each sample, approximately 10–15 mg was used for DNA isolation with a DNAeasy Blood & Tissue Kit as specified by the manufacturer (Qiagen, Inc.). Final DNA elutions were suspended in 400 µl of 10 mM Tris-HCl, pH 8.0. Samples were manually arrayed into three 96-well master plates. Five replica plates were made from each master plate, with 50 µl of DNA in each well. One set of three plates containing the 199 samples was analyzed using PCR amplified microsatellites as described below.

### Microsatellite analysis

We used 32 microsatellite loci developed by Mackiewicz *et al*. [16]. The PCR amplifications and genotyping were carried out as described therein, except that alleles in the present study were separated on a capillary instrument (GA3100) and their sizes were determined using GeneMapper software ver. 4.0 (both from Applied Biosystems).

Genetic differences between individuals were estimated using the *D*_PS_ distance metric [76] based on the proportion of shared alleles. Values of *D*_PS_ can range from zero (indicating that the compared individuals are identical) to one (when no alleles are shared). With 32 genotyped loci, a genetic distance of 0.0156 corresponds to any case in which only one allele is distinct. *D*_PS_ distances were calculated in MSAnalyser [77], and their matrices were further processed through module NEIGHBOUR of the Phylip package (version 3.573c, [78]) to obtain neighbor-joining or UPGMA phenograms. Graphical representations of these phenograms were produced using TREEVIEW (version 1.6.6, [79]).

### Genotype retesting

We initially genotyped 199 fish samples representing 51 named “clones”. The initial results indicated that certain laboratory stocks were misidentified or mixed-up. The most common were cases of mislabeling when all replicate fish of a certain stock should have belonged to a different stock based on microsatellite genotype profile. There were also several cases of mix-ups when some replicate fish of certain lab stocks represented that stock correctly, whereas others should have belonged to a different stock. To verify the initial results, 51 samples were retested, either from previously isolated DNA or from additional resubmitted samples, thus bringing our total sample size of genotyped individuals to 250. Upon completion of the tests, 39 samples were removed from further consideration because they represented obvious labeling mistakes or other mix-ups in stock identification (Table S1). Finally, four samples provided in additional submissions were added to the dataset to replace the mistaken ones. Thus, our final genetic analysis entailed 164 samples representing 42 stocks (Dataset S1).

### Mitochondrial DNA sequences

We sequenced a total of 2946 nucleotide positions from three mitochondrial regions (as described in [21]) in selected individuals representing 10 of the laboratory stocks. Alignment of the sequences was conducted with ClustalW [80] and verified visually. Kimura's two-parameter genetic distances (*K2P*) were calculated and a neighbor-joining tree was constructed using Mega3 [81].

## Results

We identified a total of 21 distinct clonal lines in our genetic assays of 164 individuals, with an average of 7.6 tested fish belonging to each such lineage. Some of the stock lines had been maintained in only one laboratory whereas others were received from as many as five different laboratories (Table 1).

### Intraline variation

**Figure 1:**
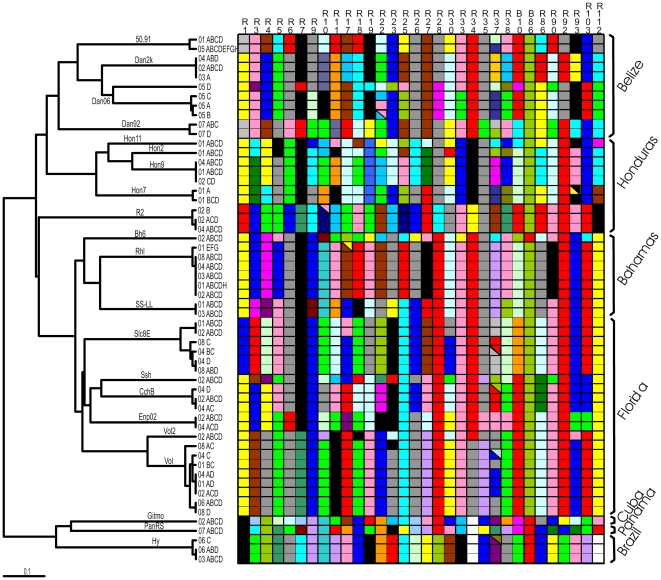
Summary of intraline and interline genetic variation in laboratory stocks of *Kryptolebias marmoratus*. UPGMA phenogram showing relationships among lines is based on *D*_PS_. Names of the laboratory lines are shown along branches of the tree. Each terminal node name consists of two parts: a number indicating the laboratory of origin (as explained in Table 1); and letters that indicate replicate individual fish from that source. Genotypes at 32 microsatellite loci (arranged in columns) are shown for each node with different colors representing different alleles. Homozygous and hererozygous genotypes are indicated by uni-coloured and bi-colored cells, respectively. Major source regions of the laboratory lines are outlined by braces.

One objective of our study was to assess the level and source of genetic variation within each laboratory line. Such knowledge could be crucial in any experiment that requires the use of genetically identical individuals. Eleven lines proved to display zero within-individual heterozygosity and no variation among specimens, so these lines can be classified as truly isogenic (i.e., composed of “clonemates”). However, within each of the remaining 10 lines, not all specimens were genetically identical (Table 2; Figure 1). In seven of these latter lines, the differences between individuals were limited to one locus. In this group were cases in which various individuals were distinct at both alleles at that locus and other cases in which only one allele was distinct (i.e., at least one individual of the line was heterozygous). Finally, for the remaining three lines, differences between individuals occurred at multiple loci, as follows: in line *Vol* (represented by 17 specimens from five laboratories, individuals differed at up to two loci; in line *Slc8E*, specimens differed at up to three loci; and in line *Dan06*, individual *D* had distinct alleles at 13 of 32 loci (*D*_PS_ = 0.406) when compared to the three other individuals from that line which were distinct from one another at either one or two alleles at only one locus.

**Table 2:** Cases of polymorphic loci in *Kryptolebias marmoratus* lines.

Line	Heterozygotes present along with homozygotes	Homozygous polymorphism (two alleles present, but only in homozygous condition)
*50.91*		*R37*
*Dan92*		*R37*
*Hon7*	*R93*	
*Rhl*	*R17*	
*Slc8E*	*R37*	*R17, R30*
*CchB*	*R37*	
*Vol*	*R37* (3 alleles)	*R23*
*R2*	*R10*	
*Hy*	*R37*	
*Dan06* a	*R22* (3 alleles)	12 loci

a This line may have originated from two progenitors.

Noteworthy, locus *R37* was frequently implicated as responsible for intraline variation; altogether we found six lines in which individuals were distinct for one or both alleles at this locus (Table 2). Furthermore, one line (*Vol*) carried three alleles at R37.

### Interline variation

Another goal of our work was to estimate genetic relationships among available laboratory lines. Such knowledge should be helpful for experiments in which an investigator requires specimens with particular genetic backgrounds. The assayed lines proved to encompass considerable genetic diversity (Figure 1). Divergence values between lines ranged from *D*_PS_ = 1 (no alleles in common, for example between *Hy*, *PanRS*, and *Gitmo* versus some of the remaining lines) to *D*_PS_ = 0.22 (78% of alleles in common, between *Vol* and *Vol2*). In general, lines originating from the same geographic region tended to cluster together in the genetic phenogram. For example, multiple lines from the Bahamas, Florida (western and southern), Belize, and Honduras (except *R2*) each formed respective genetic clusters. Moreover, this “pheno-geographic” pattern at microsatellite loci was further supported in a combined analysis of laboratory lines and specimens collected directly from the wild (see Figure S1, which also incorporates earlier datasets from Tatarenkov *et al*. [20], [21]).

**Figure 2:**
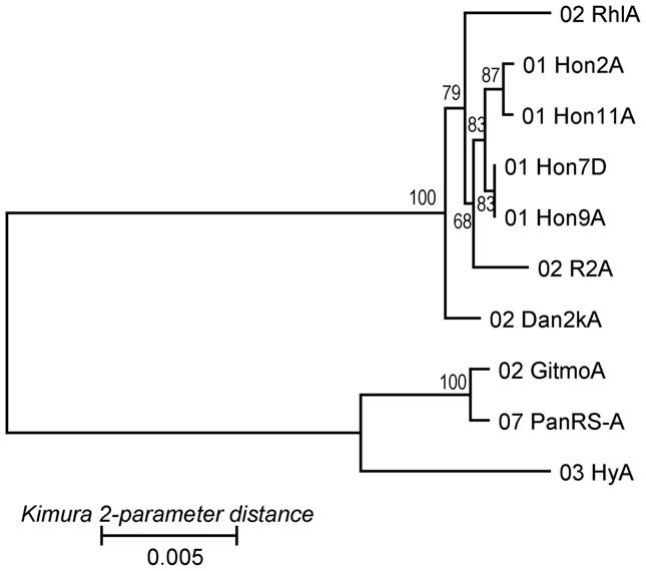
Neighbor-joining tree of selected laboratory stocks of *Kryptolebias marmoratus* based on 2,946-bp mtDNA sequences. Names of the laboratory lines are preceeded a number indicating the laboratory of origin (as explained in Table 1); letters at the end indicate replicate individual fish from that source. Bootstrap support values are shown along the nodes. Placements of these lines in the larger mtDNA that includes 136 fish from Caribbean and Brazil are shown in Figure S2.

Lines *Hy*, *Gitmo*, and *PanRS* proved to be of special interest because they clearly clustered with *K. ocellatus* rather than with *K. marmoratus* in the genetic phenograms (Figures S1 and S2). Previous phylogenetic work [82], [21] had shown that *K. ocellatus* is the sister-species (closest living relative) to *K. marmoratus*; its geographic range is poorly known, but specimens have been collected mostly in southern Brazil. With respect to the laboratory lines of “*K. marmoratus*” currently under consideration, *Hy* proved to be very similar to samples of *K. ocellatus* from Guaratiba, Brazil both at microsatellite loci (*D*_PS_ = 0.07–0.14) and in mitochondrial (mt) DNA genotype (which was indeed identical to the most common mtDNA haplotype in *K. ocellatus*). Similarly, the *PanRS* and *Gitmo* lines of “*K. marmoratus*” were genetically closer to *Hy* at microsatellites (*D*_PS_ = 0.67–0.73) and in mtDNA sequence (*K2P* = 1.1–1.2%) than they were to the other *K. marmoratus* samples examined (*D*_PS_ = 0.84–1.00 and *K2P* = 3.4–3.9%; Figures 2, S1, and S2).

## Discussion

Because of its unique biology, *K. marmoratus* has become an organism of choice for several lines of research. This small cyprinodontid killifish has a tropical and subtropical New World distribution from southern Florida to Brazil, where it inhabits estuarine and shallow intertidal locations with Red Mangroves [83]–[85], [36]. The species shows several adaptations to semi-terrestrial life [86], [87], often utilizing crab burrows and driftwood boreholes as refugia during periods of low tide [88]–[90]. However, the most unique feature of *K. marmoratus* is its reproductive biology. Natural populations are androdioecious, meaning that they consist mostly of self-fertilizing hermaphrodites but also include pure males that occur at varying frequencies in different populations [91], [11], [92], [12], [17]. Hermaphroditic individuals possess an ovotestis that utilizes normal meiotic division for spermatogenesis and oogenesis, which take place within spermatogenic and ovogenic tissues that are physically interwoven [70], [72]. The fertilization events for most eggs occur inside the fish at the time of ovulation [91]. The occasional outcross events presumably occur when a hermaphrodite sheds a few unfertilized eggs to the outside where they may be externally fertilized by sperm from a male individual.

### Genetic Inventory of Laboratory Stocks

Our genetic survey has demonstrated that cross-contamination and/or incorrect assignment of the stocks maintained in various laboratories is a serious issue. We determined that 39 of 199 individuals (20%) had an incorrect assignment (meaning that a fish that had been designated as belonging to one clone actually belonged to a different genetic lineage). For example, among the five presumably different “clones” provided by one laboratory, only two clones actually were present, and, furthermore, one of them was contaminated in the sense that it included both correctly identified and misidentified individuals. Such laboratory mix-ups were widespread and they have the potential to cause serious problems in any biological experiment in which the researcher erroneously assumes that he or she is using particular clonemate animals. For example, an experimenter might fail to acknowledge or accommodate true genetic differences between lines or might treat individuals of the same line as if they belong to different lines (leading to potential difficulties in experimental replicability or repeatability within a lab); and, if the mislabeled lines are used by multiple investigators, then problems of reproducibility across labs could be encountered as well.

### Intraline variation

Nearly half of the supposedly clonal lines sent to us for genetic inventory proved to show at least some between-individual genetic heterogeneity. In the extreme, in line *Dan06*, up to 41% of loci showed distinct alleles in pairwise comparisons between specimens. There are two explanations for such heterogeneity: segregation of polymorphisms that were present in the progenitor, and *de novo* mutations. Unfortunately, our conclusions on sources of heterogeneity must remain speculative due to the fact that none of the laboratories kept explicit pedigree records. Furthermore, even the previous exchanges of fish between laboratories were not recorded in a pedigree format, making it impossible to trace the origin of the genetic variability that we now observe. Despite such limitations, there is strong evidence that most of the detected heterogeneity was caused by *de novo* mutations, as discussed below.

Line *Dan06* was collected in 2006 and shows high intraline diversity, but the other nine lines show low intraline heterogeneity (i.e., at 1–3 loci only). The most recently founded of these lines was established at least seven years ago, whereas the others were established between 15 and 19 years ago. Killifish are capable of producing 3–4 generations per year [93], which translates to >20 generations for the most recently founded line and as many as 60 lab generations for the earliest established lines. Even if we conservatively estimate one generation per year, a sufficient number of generations of strict selfing has transpired to make it highly unlikely that a progenitor's few variable loci have retained heterozygosity continuously to the present time. Nevertheless, we found heterozygosity in seven of nine lines, or in seven of the 12 locus X line combinations (Table 2). Such cases can be explained by post-formational mutations that introduced new alleles to a line, but with insufficient time having subsequently transpired for the completion of allelic segregation to homozygosity. This conclusion holds even if the most variable locus (*R37*) is not considered. Disregarding *R37*, there are three loci at which heterozygosity is present (each in a separate line) and three other loci at which segregation has gone to completion. Further evidence for recurrent mutations is the *prima facie* observation of three alleles at locus *R37* in line *Vol*, where at least one post-origin mutation must have taken place (because the diploid progenitor could not have carried more than two alleles).

We thus conclude that genetic variation found in nine lines likely arose during the course of laboratory breeding and that the founding progenitors of those lines were highly inbred (homozygous). Only lines *Slc8E* and *Vol* were found to be variable at more than one locus, and although some of that variation could trace back to the progenitor, it is also plausible that most or all of it results from *de novo* mutations in the laboratory, considering that the lines were maintained for about 15–60 generations. If we assume that variation at *Slc8E* did indeed arise by *de novo* mutations in the laboratory during descent from the progenitor, then the mutation rate can be roughly estimated asµ  =  # mutations/(2* #generations*# loci)  = 3/(2*[15 to 60]* 32). The mutation rates thus estimated range from 7×10^−4^ to 3×10^−3^ per locus per generation, which are standard mutation rates for microsatellite loci in other species [94]. [Note: Our estimates for *K. marmoratus* nevertheless are crude. On the one hand, the mutation rates may be underestimated because individuals included in the analysis do not necessarily trace back directly to the original founder, but instead may have shared more recent ancestors. On the other hand, inclusion of the hypermutable *R37* would have yielded an inflated estimate for the mutation rate at a more typical locus.]

Line *Dan06* presents a conundrum because individual *D* had alleles different from those of individuals *A, B,* and *C* at 13 of the 32 loci surveyed and such divergence cannot readily be explained by an accumulation of mutations in the mere five years that this stock has been maintained in the lab. Thus, provided that no inadvertent stock mix-ups occurred during breeding, the intraline diversity in *Dan06* must be due to heterozygosity present in the progenitor. However, we actually suspect that this heterogeneity could be due to breeding mistakes, for two reasons. First, it is suspicious that no variation was detected among individuals *A, B,* and *C*. If the *Dan06* progenitor was indeed heterozygous at many loci, we would expect greater diversity among its descendants (although the near-clonality of *A, B,* and *C* could be explained if these specimens actually trace back to a more recent common ancestor). The second argument stems from considerations about the high magnitude of intraline variation, and in particular the high divergence of *D* from *A*, *B*, and *C*. For individuals of a purely selfing line to be distinct at 41% of loci, the progenitor should in theory be heterozygous at about 82% of loci. Although we have no data on levels of *H* in the population where *Dan06* was collected (near Dangriga, Belize), only a few individuals from a nearby population (Twin Cays, Belize) showed heterozygosity values above 70% [17]. Twin Cays is exceptional among *K. marmoratus* populations in that it has a high frequency of males, and, as a result, a high frequency of outcrossing. Males are infrequent in Dangriga [15], and, thus, the origin of individuals via outcrossing must be rarer than at Twin Cays, meaning that the chance of collecting a progenitor of extremely high *H* would be low. Thus, we consider it quite likely that *Dan06D* and *Dan06ABC* originated from different progenitors. In any event, whatever the cause of divergence between *Dan06D* and *Dan06ABC*, the most important point is that these laboratory lines should henceforth not be lumped into one “clonal” stock.

### High variation at *R37*

Locus *R37* was a significant contributor to the intraline variation presumably attributable to *de novo* mutations. Indeed, if tallied by the number of line-by-locus combinations (see Table 2), *R37* accounted for 50% of all cases of intraline variability. Given this fact, the mutation rate at *R37* might be as much as 30X greater than those at the other loci. Another way to consider this possibility is as follows. At *R37*, specimens from two lines (*50.91* and *Dan92*) were homozygous for different alleles whereas heterozygotes as well as homozygotes were present in the other four lab lines. Assuming that variation at *R37* is neutral, then the mutations in the heterozygous lines may have occurred quite recently (perhaps within the last eight generations, taking into account the expected two-fold decay of heterozygosity per generation with selfing and an average of eight replicas per line). If the mutations at *R37* did indeed take place within the last eight generations in the 21 examined lines, then the mutation rate at *R37* could be as high as 3×10^−2^ per generation. Interestingly, *R37* had at least 2X more repeats than the other loci (see Table 1 in Ref [16]), a molecular feature that might have promoted its higher mutation rate. However, a slower than expected decay of heterozygosity might also explain the high variation at *R37*. The decay of heterozygosity could have been decelerated by outcrossing, but outcrossing would affect all loci and thus cannot explain the uniqueness of *R37*. Finally, a slowed decay of heterozygosity might also be caused by any selective pressure that affords a fitness advantage to heterozygotes. A search of GenBank reveals that the flanking regions of *R37* have high sequence similarities (82% and 75%, respectively) to the hepsidin precursor locus in a rockfish (GenBank accession EU555381) and to a non-coding region that lies within MHC class I region of the Japanese medaka (BA000027). Hepsidin is known to have anti-fungal activity, and MHC plays an important role in the immune response. In addition, the MHC I locus of *K. marmoratus* was also found to be highly heterozygous and diverse in allelic composition, as compared to other highly homozygous loci, among wild-type populations surveyed [95]. These facts give some credence to the possibility that *R37* might be linked to a locus subjected to overdominant selection.

### Interline diversity and its broader implications

Some unexpected findings came from lines *Hy*, *Gitmo*, and *PanRS*. The origin of the *Hy* line was unknown, but genotypically this lineage was found to be very similar (*D*_PS_ = 0.07–0.14) to specimens collected in Brazil that may represent another species, *K. ocellatus* [21], [22]. The mtDNA sequence data confirmed such an affinity (Figure S2), because the *Hy* haplotype was identical to the most common mtDNA haplotype in fish from Guaratiba, Brazil. The high similarity of *Hy* and Guaratiba samples at microsatellite loci and in mtDNA sequence suggests that *Hy* may have been collected in this same geographic region as well. Furthermore, *Gitmo* and *PanRS* from Cuba and Panama, respectively, were rather similar to *Hy* (and to *K. ocellatus* from Brazil) at both microsatellites and mtDNA (Figures S1 and S2). This finding opens some exciting prospects regarding the evolution of the *K. marmoratus*/*K. ocellatus* clade. First, it indicates that the two lineages live in close proximity in the Caribbean, and that their ranges may overlap. Mapping of the distribution of *Kryptolebias* clades in the Caribbean is needed to find out about such an overlap. Second, it appears that the two lineages may have come into proximity after some period of separation (as opposed to accumulating differences via isolation-by-distance). This interpretation is suggested by the fact that *Gitmo* and *PanRS* are geographically close to the other Caribbean samples yet are genetically related more closely to the Brazilian collections. Third, hybrid progeny between *PanRS* and *Dan92* apparently are viable [96]. Thus, if secondary contacts exist in nature, they might present good opportunities to address the possibility and consequences of natural hybridization between the *marmoratus* and *ocellatus* lineages in future studies.

### Conclusions and suggestions

1) We found that cross-contamination of existing laboratory stocks of *K. marmoratus* is a serious issue. Thirty-nine among 199 individuals (20%) had been labeled incorrectly. Thus, we suggest that all laboratories working with *K. marmoratus* update their inventories by re-assigning any mislabeled stocks and replacing them with corrected lines. The husbandry protocols should also be revised so as to minimize accidental cross-contamination of lines. We further suggest the establishment of a consortium or “stock center” for *K. marmoratus* so that researchers can share genotypically defined lines that will facilitate future research. The authors at Valdosta State University are currently initiating such a stock center for *K. marmoratus* based on the data presented here.

2) With one exception (*Dan06*), individuals within a line showed genetic differences at three microsatellite loci, at most. *De novo* mutations that arose during laboratory propagation probably account for most of this variation, but genetic segregation from a heterozygous progenitor remains an alternative explanation in some cases. In at least one case (involving line *Dan06*) genetic variation within a “clonal” stock seems most likely to be the result of segregation in lineages that originated from two different progenitors.

3) Overall, it appears that the presence of heterozygous individuals in natural populations was not the source of intraline differentiation. This interpretation can be rationalized by the fact that progenitors typically were collected in populations with low frequencies of males and, hence, presumably low outcrossing rates. Nevertheless, the practice of using wild progenitors as founders of clonal lines without checking their genetic composition should be discouraged. If genetic screening is not feasible, we suggest propagating the wild progenitor for about ten generations, using only one individual from each generation as a parent for the next, and then using the F10 generation individual to establish an inbred stock of “clonal” individuals. By this procedure, the original heterozygosity will have been reduced to 0.195% by the tenth generation (and to 0.006% if carried out to 15 generations), assuming a 50% loss in heterozygosity per generation.

4) One locus (*R37*) appeared to have a much higher mutation rate than the other loci, having mutated independently in six different lines, and doing so twice in one lineage. In general, however, we do not consider polymorphism at *R37* to be grounds for abandoning these otherwise “clonal” lines for experimental research. Nevertheless, our findings carry a broader message; perhaps some loci with similarly high rates of mutation may have a large phenotypic effect, in which case the accumulation of mutations through time will lead to divergence of homozygous individuals within particular inbred lines at selectively important traits. To prevent this kind of outcome in experiments in which homozygosity as well as isogenicity is crucial, we suggest using *K. marmoratus* individuals that have shared as recent an ancestor as is feasible.

5) Established “clonal” stocks of *K. marmoratus* proved to represent mostly homozygous collections from various geographic areas including Florida, Bahamas, Honduras, Panama, Cuba, and, perhaps, Brazil. This geographic breadth is reflected in considerable genetic divergence between laboratory lines, with some such lines sharing no alleles at 32 microsatellite loci and showing mtDNA sequence divergences as high as 4.1%. This wealth of genetic diversity in laboratory stocks of *K. marmoratus* provides rich material for experimentalists who might wish to compare, for example, the performances of distinct clones in particular ecological settings or to test various hypotheses about outbreeding depression in crosses between diverse lines.

## Supporting Information

Table S1 Summary of samples removed based upon genotype.
(0.02 MB XLS)

Figure S1 Microsatellite-based neighbor-joining tree showing positions of 22 individuals representing 21 lines (line Dan06 represented by 2 individuals) among fish specimens collected in nature (using datasets from [20], [21]). Laboratory lines are shown in red.
(0.04 MB PDF)

Figure S2 Positions of some laboratory lines in a larger mtDNA tree. Additional samples used in this tree are those from [21]. Laboratory lines are highlighted in yellow. Lines PanRS (Panama) and Gitmo (Cuba) cluster with *Kryptolebias ocellatus*.
(0.08 MB PDF)

Dataset S1 Microsatellite genotypes of 164 individuals from 42 laboratory stocks representing 21 lines of *Kryptolebias marmoratus*.
(0.09 MB XLS)
