# Supplementary figures and images for: Genetic Composition of Laboratory Stocks of the Self-Fertilizing Fish Kryptolebias marmoratus: A Valuable Resource for Experimental Research
Source: PLoS One. 2010 Sep 22;5(9):e12863. doi: 10.1371/journal.pone.0012863 (PMC2943930; doi:10.1371/journal.pone.0012863)

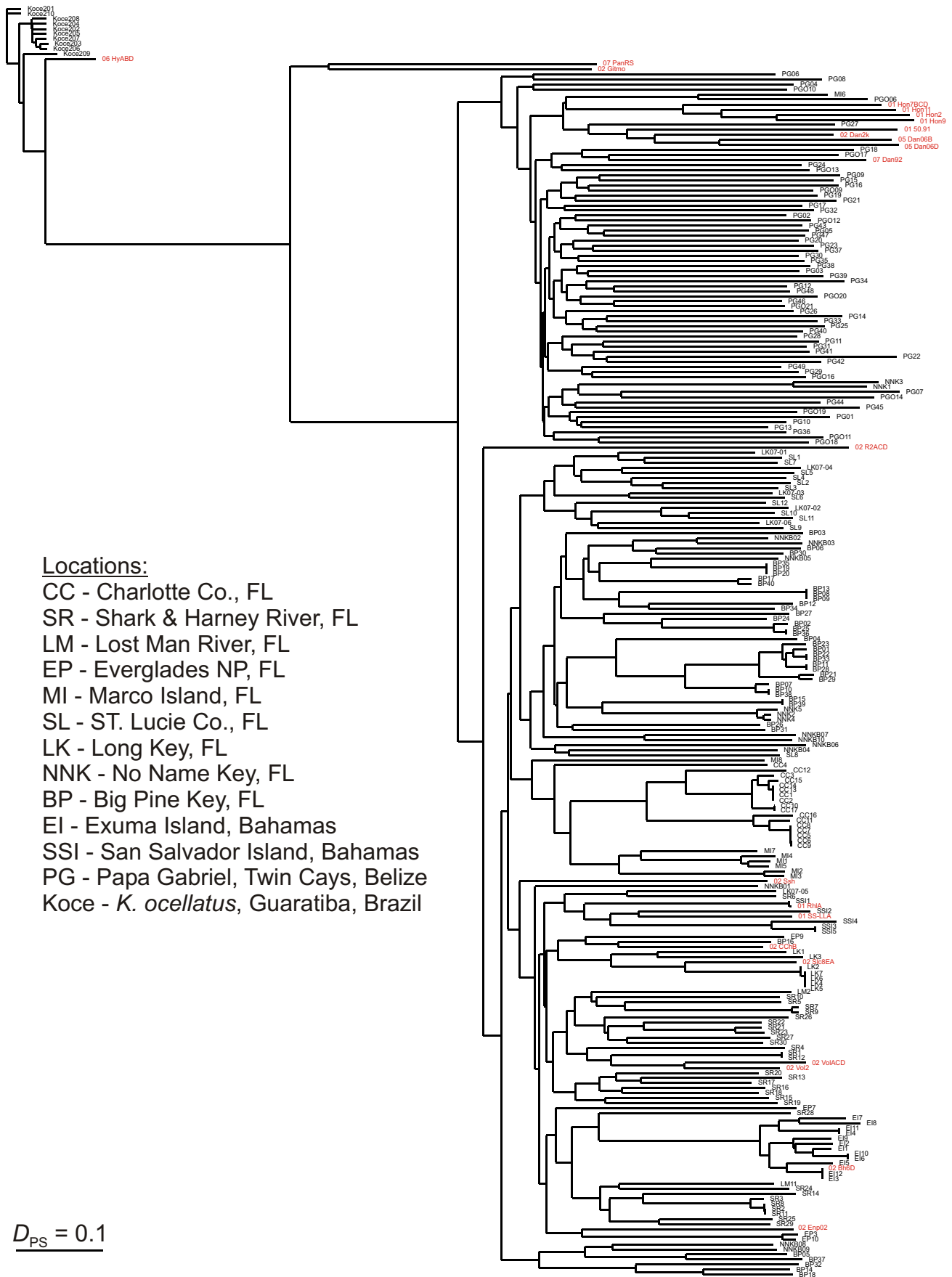

Figure S1

Supplement: Figure S1 — Microsatellite-based neighbor-joining tree showing positions of 22 individuals representing 21 lines (line Dan06 represented by 2 individuals) among fish specimens collected in nature (using datasets from [20], [21]). Laboratory lines are shown in red. (0.04 MB PDF) [file pone.0012863.s002.pdf]

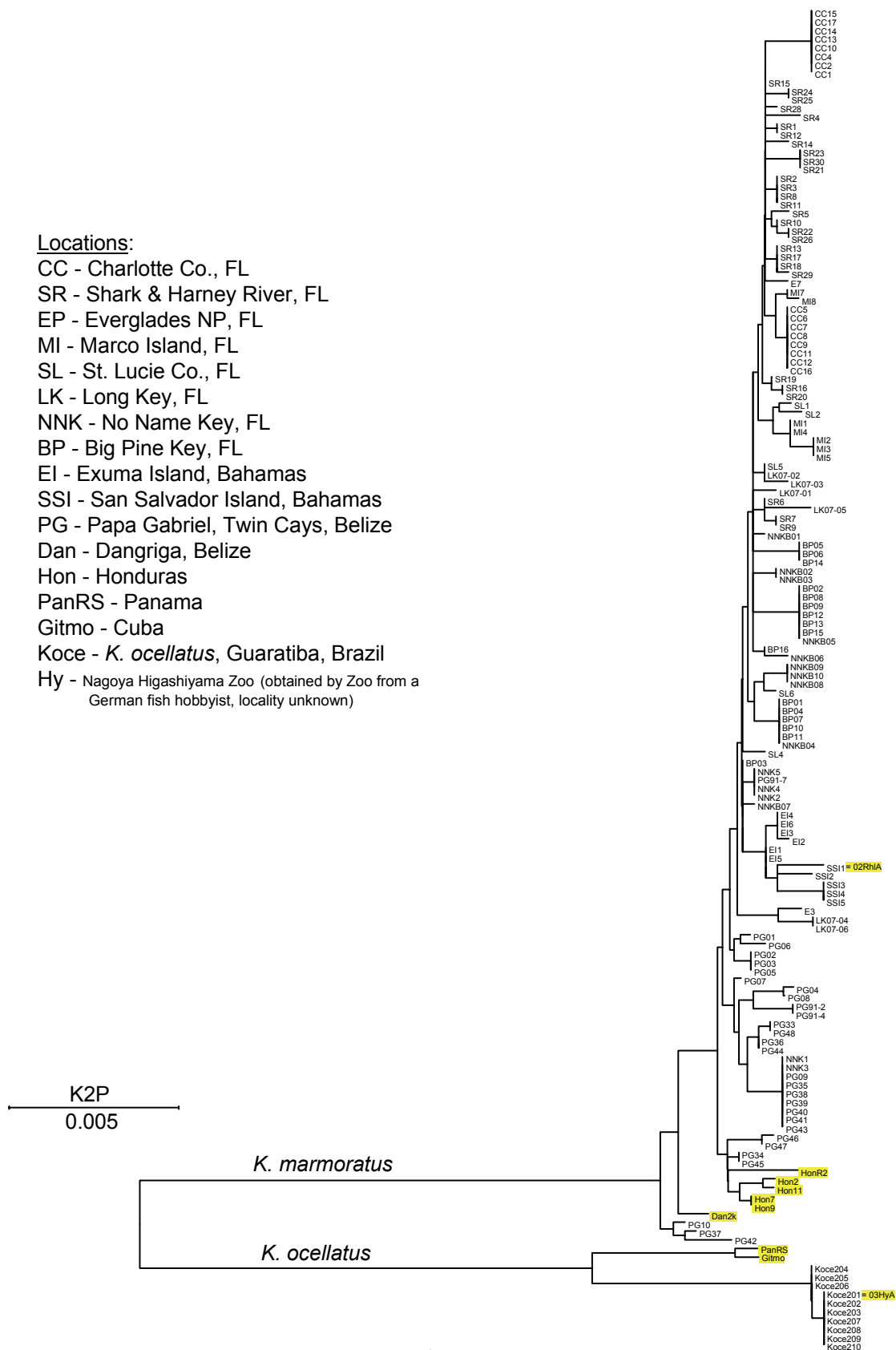

Figure S2

Supplement: Figure S2 — Positions of some laboratory lines in a larger mtDNA tree. Additional samples used in this tree are those from [21]. Laboratory lines are highlighted in yellow. Lines PanRS (Panama) and Gitmo (Cuba) cluster with Kryptolebias ocellatus. (0.08 MB PDF) [file pone.0012863.s003.pdf]
